# Identification of receptor-binding protein and host receptor of non-lytic dsRNA phage phiNY

**DOI:** 10.1128/spectrum.01467-24

**Published:** 2024-10-22

**Authors:** Guoqing Ding, Hongmei Liu, Jing Lan, Tianbao Qian, Yan Zhou, Tongyu Zhu, Tingting Zhang

**Affiliations:** 1 School of Public Health, the key Laboratory of Environmental Pollution Monitoring and Disease Control, Ministry of Education, Guizhou Medical University, Guiyang, China; 2 Engineering Research Center of Health Medicine biotechnology of Institution of higher education of Guizhou Province, School of Biology and Engineering (School of Modern Industry for Health and Medicine), Guizhou Medical University, Guiyang, China; 3 Shanghai Medical College, Fudan University, Shanghai, China; 4 Shanghai Institute of Phage, Shanghai Public Health Clinical Center, Fudan University, Shanghai, China; Universidad Nacional Autonoma de Mexico - Campus Morelos, Cuernavaca, Mexico

**Keywords:** dsRNA phage phiNY, receptor-binding protein, host receptor, *M. aerodenitrificans*strain LH9

## Abstract

To date, complete genome sequences of 14 double-stranded RNA (dsRNA) phages are available, and studies have shown that the host range of dsRNA phages is limited. The hosts of most dsRNA phages belong to the genus *Pseudomonas*. However, the dsRNA phage phiNY, which has a non-lytic life cycle, was isolated from *Microvirgula aerodenitrificans*. Currently, the interaction between dsRNA phage phiNY and its host bacteria is unclear, which is not beneficial to a comprehensive understanding of dsRNA phage biology and the exploitation of dsRNA phage with non-lytic life cycle for biomedical applications and others. Phage adsorption is a crucial step through the interactions between receptor-binding protein (RBP) of the phage and its receptors to initiate the infection process, which dictates host range specificity. Thus, we identified the RBP and host receptor of phiNY. Through homology alignment, amino acid sequence similarity analysis, and the phylogenetic tree analysis, orf11, located in the M-segment of dsRNA phage phiNY, encodes a putative RBP. We further performed the whole-cell enzyme-linked immunosorbent assay (ELISA), western blotting assay, and indirect immunofluorescence assay and demonstrated that this orf11 is an RBP. Finally, using affinity chromatography, ELISA, and dynamic light scattering, we identified lipopolysaccharides (LPSs) on the surface of the host *M. aerodenitrificans* strain LH9 as host receptors involved in the adsorption of the dsRNA bacteriophage phiNY and observed the state of phiNY RBP after combining with LPS by atomic force microscopy. These results will guide future studies on phage-host interaction in a dsRNA phage with a non-lytic life cycle.

**IMPORTANCE:**

The interactions between the lytic dsRNA phages and their host receptors have been clarified in previous studies. However, the interaction between the dsRNA phage phiNY (which has a non-lytic life cycle) and its host receptors during the dsRNA phage adsorption process was unknown. Here, we found that phiNY uses the orf11 protein as a receptor-binding protein (RBP). In addition, we found that this orf11 recognizes lipopolysaccharide from the host bacterium *Microvirgula aerodenitrificans* strain LH9 as a speciﬁc receptor. These results suggest that phiNY, like lytic dsRNA phages, uses an RBP to bind to a similar host receptor (i.e., lipopolysaccharide). Determining the interaction between the dsRNA phage phiNY and its host receptors will help to elucidate the mechanisms underlying the phiNY non-lytic life cycle and enhance our understanding of its infection mechanism.

## INTRODUCTION

Bacteriophage adsorption to its host is the critical first step for a successful bacteriophage infection, relying on the highly specific interaction between a receptor-binding protein (RBP) and its host receptor. This adsorption is generally depicted as a three-step process: initial contact, reversible binding, and irreversible attachment (1, 2). The interaction between a phage’s RBP and its host receptor determines the phage host range and can provide crucial information for understanding the anti-phage defense mechanisms of phage treatments (1, 3). Engineering RBPs can extend the phage host range to create efficient and rapid phage cocktails to target human pathogens for therapeutic application (4–6). In addition, engineering RBPs can also be used as rapid diagnostic tools. Currently, various engineering RBP detection assays have been developed for detecting *Salmonella* (7), *Yersinia* (8), *Shigella* (9), *Escherichia coli* (10), *Klebsiella pneumoniae* (11), and *Pseudomonas aeruginosa* (12).

Previous studies mainly focused on DNA phage adsorption and described their RBPs and host receptors. The RBPs of DNA phages are the specialized bio-nanomachinery that specifically recognize their host cells, penetrate the host cell surface, and eject the DNA phage genome into the host cytosol to produce new progeny phages (13). Usually, the RBPs of DNA phages are located at the distal end of the tail and mediate phage binding specifically to their receptor on the host surface, such as lipopolysaccharide (LPS), teichoic acids, transmembrane porin proteins, and even organelles (e.g., pili or flagella) (1). In contrast to previous studies on the RBPs of DNA phages, previous studies on the RBPs of dsRNA phages are insufficient and mainly involve their identification in genome organizations based on sequence similarities in relation to well-characterized systems.

The RBPs (also known as spike proteins or P3 protein complexes) of dsRNA phages, located at the lipid envelope, mediate phage binding to specific receptors such as a type VI pili or LPS (14–23). In addition, the P3 protein complex is multimeric (comprising multiple identical polypeptides), in phages phi6, phi2954, and phiNN (17, 18, 24), but is heteromeric (containing two or three different polypeptides) in phages phi8, phi12, phi13, phiYY, and phiZ98 (15, 19, 21, 25).

The full-length genome sequence of the dsRNA phage phiNY was determined in our previous study (26). It was highly similar to other dsRNA phages in terms of its genome organizations but had lower sequence similarities in relation to these other dsRNA phages (26). In addition, unlike other dsRNA phages with a lytic lifestyle, most of which infect the genus *Pseudomonas*, phiNY has a persistent infection lifestyle and was isolated from *M. aerodenitriﬁcan*. However, the RBP of phiNY and host receptors are unclear. Therefore, understanding how non-lytic dsRNA phage phiNY recognizes its specific host receptor has several important significances: (ⅰ) novel dsRNA phage RBP and host receptor might be found; (ⅱ) to improve the understanding of the diversity of dsRNA phage adsorption strategy; (ⅲ) to advance understanding of dsRNA phage evolution and their host range adaptation.

## RESULTS

### Bioinformatics analysis of the putative RBP of dsRNA phage phiNY

**Fig 1:**
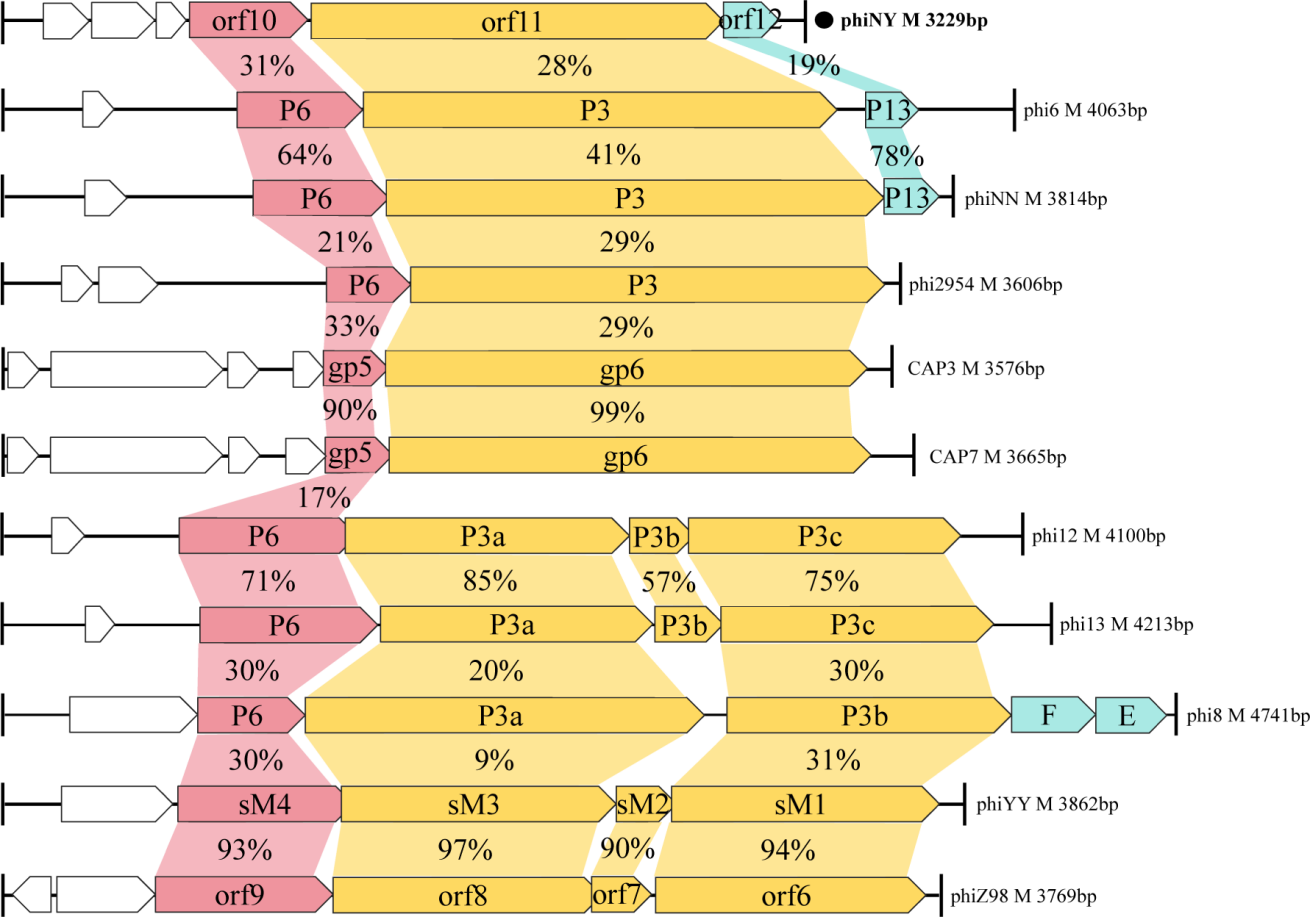
Genome maps of the M-segments of dsRNA phages showing the amino acid sequence similarities (%) of open reading frames (ORFs) of dsRNA phages based on pairwise sequence alignment using EMBOSS Needle.

The genome organizations of dsRNA phages are highly similar (27). They encode proteins (on corresponding genome segments) that function similarly (27). The L-segment encodes the core protein components (P1, P2, P4, and P7), the M-segment encodes proteins with host recognition function (P3 and P6), and the S-segment encodes the nucleocapsid shell protein (phi8 P8; as an exception), major membrane protein (P9), putative membrane morphogenetic factor (P12), and lysis protein (P5). Comparative genome analysis revealed that orf11, in the M-segment of the dsRNA phage phiNY, encodes putative P3 protein (also called an RBP; Fig. 1).

**Fig 2:**
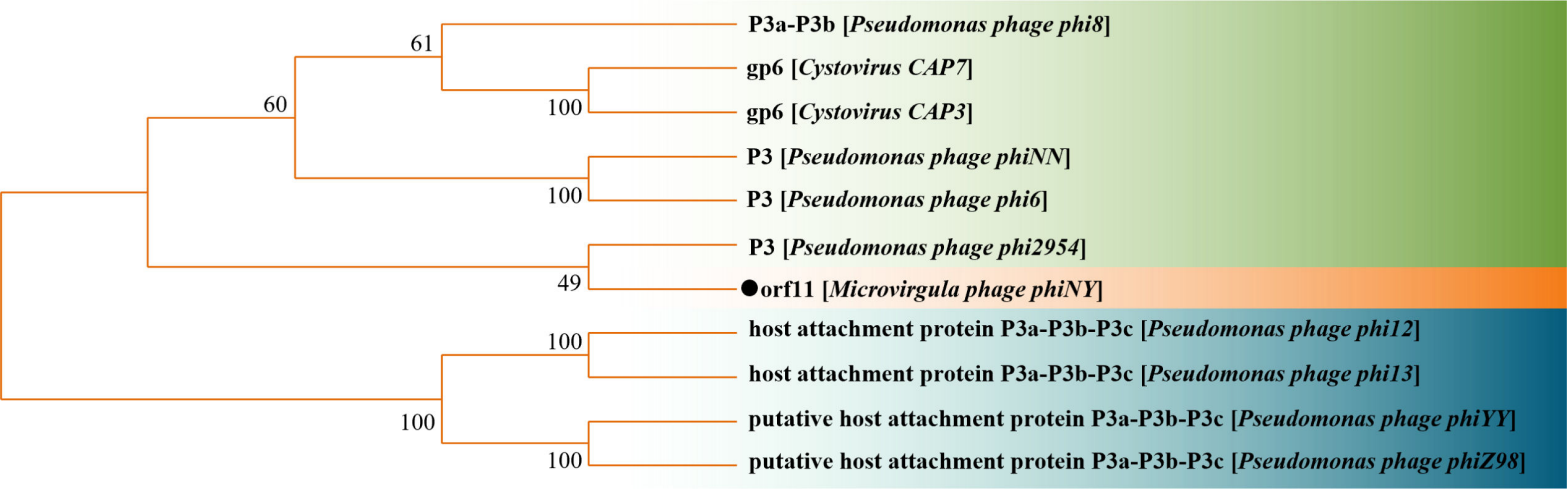
Phylogenetic trees of dsRNA phages based on amino acid sequences of putative RBPs. The tree was constructed with the neighbor-joining (NJ) method using MEGA 11.

Furthermore, we constructed the phylogenetic tree analysis between orf11 and other P3 proteins (Fig. 2). The phylogenetic trees show that orf11 and the P3 protein of phi2954 are the most related, and the P3 proteins of phages phi8, phiNN, phi6, CAP7, and CAP3 are also closely related to orf11; these phages use a type IV pilus-mediated adsorption strategy. However, orf11 and the P3 genes of the other dsRNA phages share limited similarity (31.31%–42.04%) at the nucleotide sequence level (Table 1).

**TABLE 1:** Nucleotide sequence similarities (%) between the orf11 of dsRNA phage phiNY and the P3 proteins of other dsRNA phages^*a*^

Nucleotide sequence similarities (%) between the orf11 and P3											
phage	phiNY	phiNN	phi6	phi8	phi2954	CAP3	CAP7	phiYY	phi12	phiZ98	phi13
phiNY	100	42.04	41.46	38.81	36.4	32.66	32.34	31.96	31.64	31.56	31.31
phiNN		100	44.83	38.56	37.77	33.97	34.81	32.89	33.18	33.67	33.33
phi6			100	39.43	40.02	32.65	33.20	32.54	32.62	33.57	29.32
phi8				100.00	38.35	36.84	37.20	40.52	38.82	41.34	39.49
phi2954					100.00	31.76	31.45	32.06	31.40	33.74	31.85
CAP3						100.00	86.86	36.99	35.77	37.45	34.88
CAP7							100	38.43	37.65	38.51	36.24
phiYY								100	58.01	81.96	57.60
phi12									100	57.93	64.12
phiZ98										100	56.89
phi13											100

^*a*^ Color code: >95% = dark gray, >75% = medium gray, and >50% = light gray.

### The native state of orf11 is a multimer

The P3 protein complex is a multimer (comprising multiple identical polypeptides) in phages phi6, phi2954, and phiNN (17, 18, 24). However, the P3 complex is heteromeric (comprising two or three different polypeptides) in phages phi8, phi12, phi13, phiYY, and phiZ98 (19, 21, 25, 28). In this study, recombinant orf11 contained 589 amino acids plus the His6-tag and had an estimated molecular weight of 65 kDa according to SDS-PAGE (Fig. S1A). Native-PAGE (Fig. S1B) and glutaraldehyde cross-linking assays (Fig. S1C) revealed that the native state of orf11 is a multimer.

### orf11 protein of dsRNA phage phiNY binds specifically to *Microvirgula aerodenitrificans* strain LH9 cells

**Fig 3:**
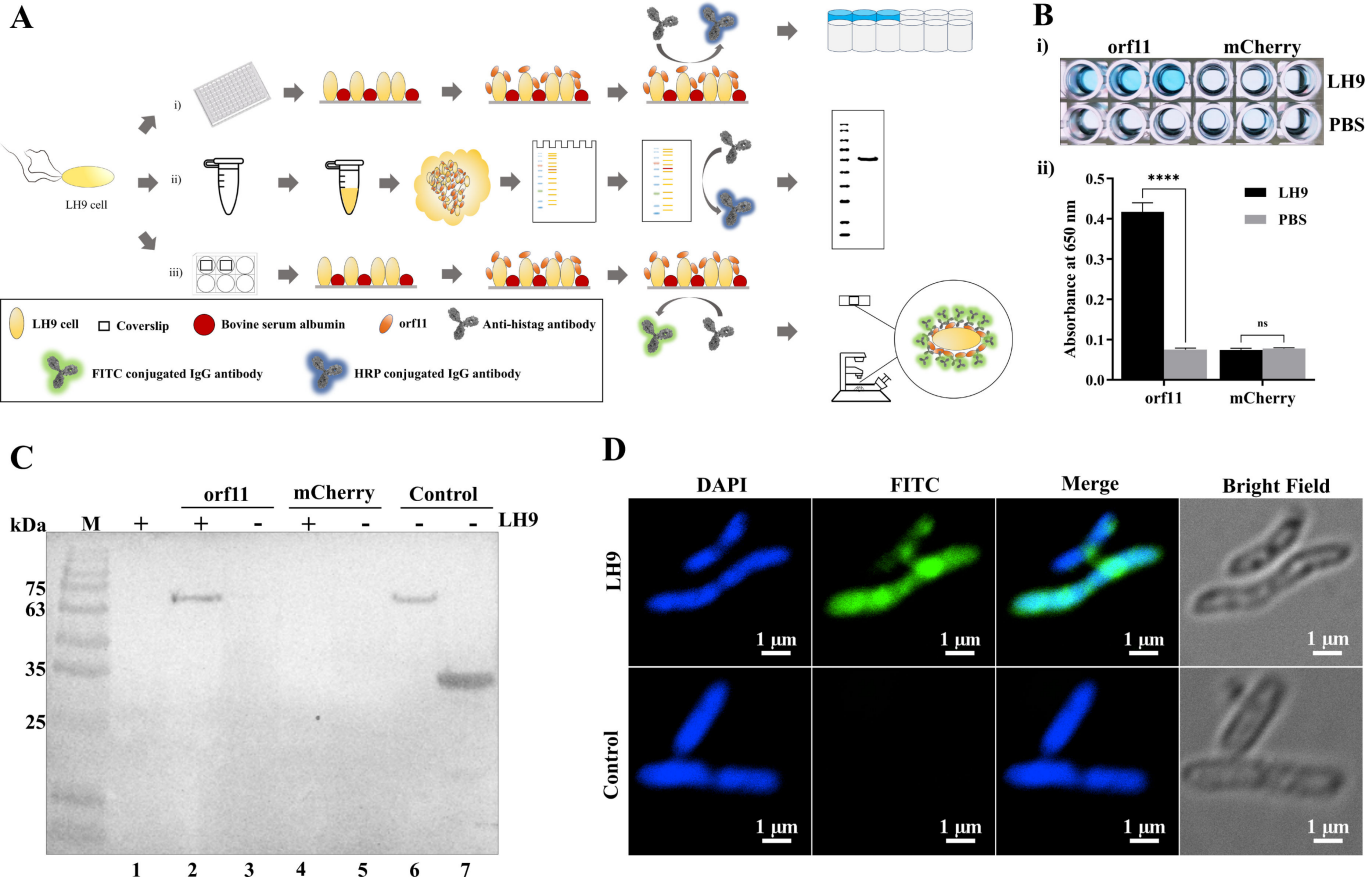
orf11 protein binds specifically to *M. aerodenitrificans* strain LH9 cells. (**A**) (ⅰ) Enzyme-linked immunosorbent assay (ELISA) procedure, (ⅱ) western blotting procedure, and (ⅲ) indirect immunofluorescence assay procedure. (**B**) Preliminary ELISA verification of orf11protein adsorption of strain LH9. (ⅰ) ELISA plate image of 3,3′,5,5"-tetramethylbenzidine (TMB) substrate after 30 min of color development. (ⅱ) ELISA preliminarily verified the increased absorbance at 650 nm, indicating binding of His-tagged orf11 protein to strain LH9, compared to the negative control (His-tagged orf11 protein plus phosphate buffered saline, PBS). The second negative control (His-tagged mCherry, a non-specific protein that does not bind to the *M. aerodenitrificans* strain LH9 cells) also led to a low signal intensity (****P < 0.0001, and ns, not signiﬁcant, Student’s *t* test). (**C**) Western blotting verification of orf11 protein binding to strain LH9. The binding of His-tagged orf11 protein was detected using monoclonal anti-His-tag antibody. Lane 1 is a negative control involving strain LH9 only. Lanes 3 and 5 are negative controls involving the orf11 protein and mCherry, respectively (without strain LH9). Lanes 6 and 7 are positive controls involving purified orf11 and mCherry, respectively (without strain LH9). (**D**) Indirect immunofluorescence assay verification of orf11 binding to strain LH9. The binding of His-tagged orf11 protein was detected using monoclonal anti-His-tag primary antibody, and fluorescein isothiocyanate (FITC) conjugated secondary antibody was used to detect the primary antibody. *K. pneumoniae* strain 11272018 (which does not bind to orf11 protein) was used as the negative control.

The binding of the orf11 protein to *M. aerodenitrificans* strain LH9 cells, which is the host strain of the dsRNA phage phiNY, was confirmed by three different assays (Fig. 3A).

First, we performed whole-cell enzyme-linked immunosorbent assays (ELISAs) to determine the binding of purified orf11 proteins to *M. aerodenitrificans* strain LH9 cells (29). His-tagged orf11 protein plus *M.aerodenitrificans* strain LH9 cells exhibited a significant increase in signal intensity compared to the negative control (His-tagged orf11 protein plus PBS; Fig. 3B). The second negative control (His-tagged mCherry, a non-specific protein that does not bind to *M. aerodenitrificans* strain LH9 cells) also led to a low signal intensity. In addition, we tested the binding of orf11 proteins to five other strains of non-host bacteria. The results showed that orf11 does not bind to them (Fig. S2), indicating that orf11 does not cross-react with other different bacterial species and binds specifically to *M. aerodenitrificans* strain LH9 cells.

Second, we performed western blotting assays. The orf11 protein was incubated with *M. aerodenitrificans* strain LH9 cells, and cell-bound orf11 protein was observed by western blotting after washing off the unbound orf11 protein. As a negative control, the mCherry protein does not bind to *M. aerodenitrificans* strain LH9 cells. The results confirmed that the orf11 protein could bind to *M. aerodenitrificans* strain LH9 cells (Fig. 3C).

Third, an indirect immunofluorescence assay was used to directly observe the interaction between the orf11 protein and *M. aerodenitrificans* strain LH9 cells. In this assay, the binding of His-tagged orf11 protein was detected using an anti-His-tag primary antibody, and FITC-conjugated secondary antibody was used to detect the primary antibody. The results showed that the combination of the orf11 protein and *M. aerodenitrificans* strain LH9 cells produced a strong FITC fluorescence signal; however, the orf11 protein and *K. pneumoniae* strain 11272018 cells (negative control that does not bind to orf11 protein) did not produce an FITC fluorescence signal, further indicating that orf11 protein specially binds to *M. aerodenitrificans* strain LH9 cells (Fig. 3D).

Together, the above results indicated that the orf11 protein is the RBP of the dsRNA phage phiNY, and it binds specifically to *M. aerodenitrificans* strain LH9 cells.

### Identification of host receptor of dsRNA phage phiNY

The dsRNA phage phiNY has a latent infection lifestyle, infecting its host bacterium *M. aerodenitrificans* strain LH9 (which does not have a mature genetic expression system) without producing phage plaques. We identified the host receptor of phiNY using three different assays.

First, affinity chromatography assays showed that purified orf11 protein of phiNY does not bind to proteins from *M. aerodenitrificans* strain LH9 cells (Fig. S3).

**Fig 4:**
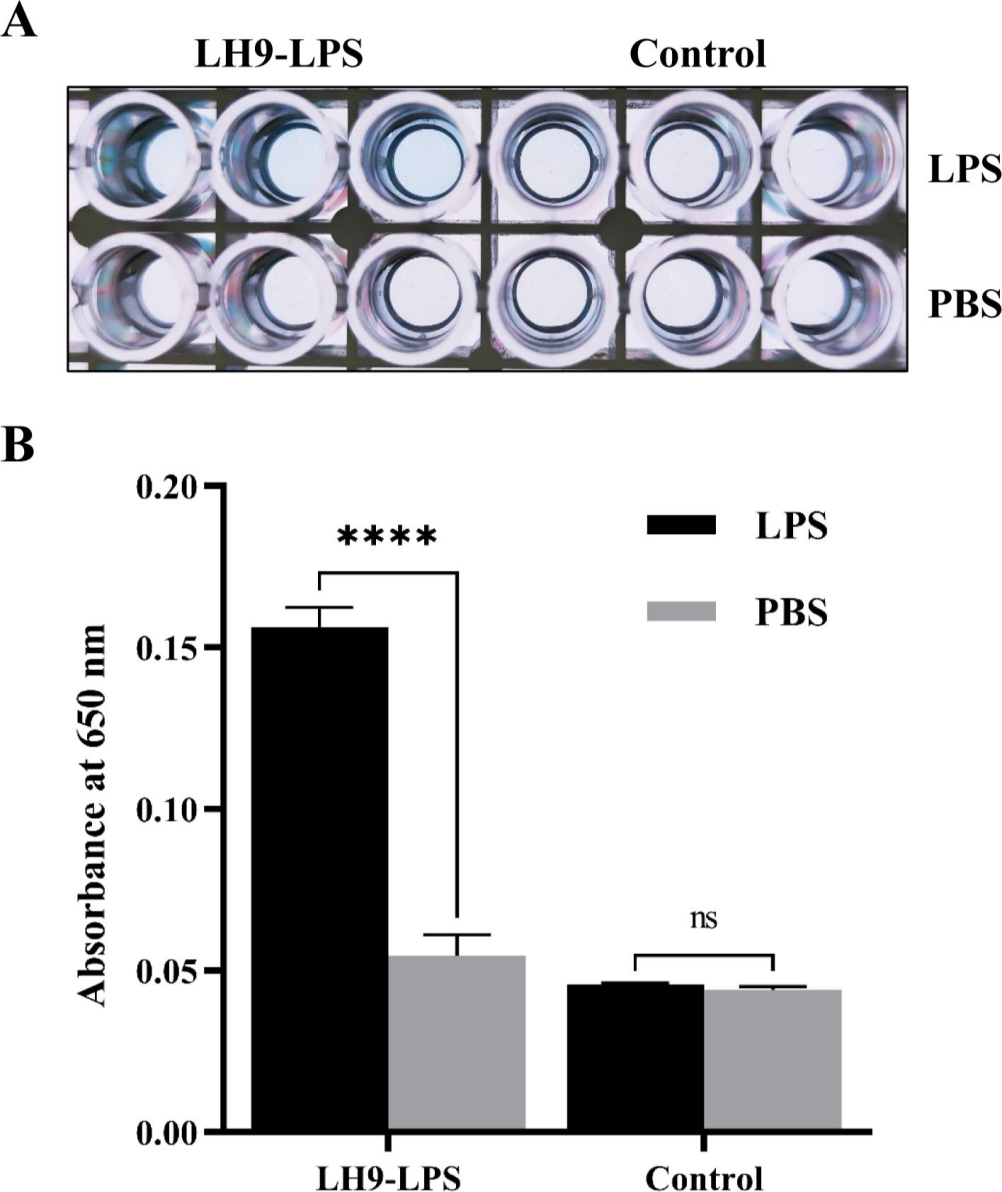
ELISA showing that the orf11 protein binds specifically to LPS from *M. aerodenitrificans* strain LH9 cells. (**A**) ELISA plate image of TMB substrate after 30 min of color development. (**B**) ELISA results of purified orf11 protein binding to extracted LPS from *M. aerodenitrificans* strain LH9 cells based on an absorbance at 650 nm. Extracted LPS from *K. pneumoniae* strain 11272018 that does not bind to orf11 protein was the negative control (****P < 0.0001, and ns, not signiﬁcant, Student’s *t* test).

Second, ELISAs showed that purified orf11 protein binds specifically to LPS extracted from *M. aerodenitrificans* strain LH9 cells (Fig. 4). Therefore, we hypothesized that purified orf11 protein most likely interacts with LPS from *M. aerodenitrificans* strain LH9 cells.

**Fig 5:**
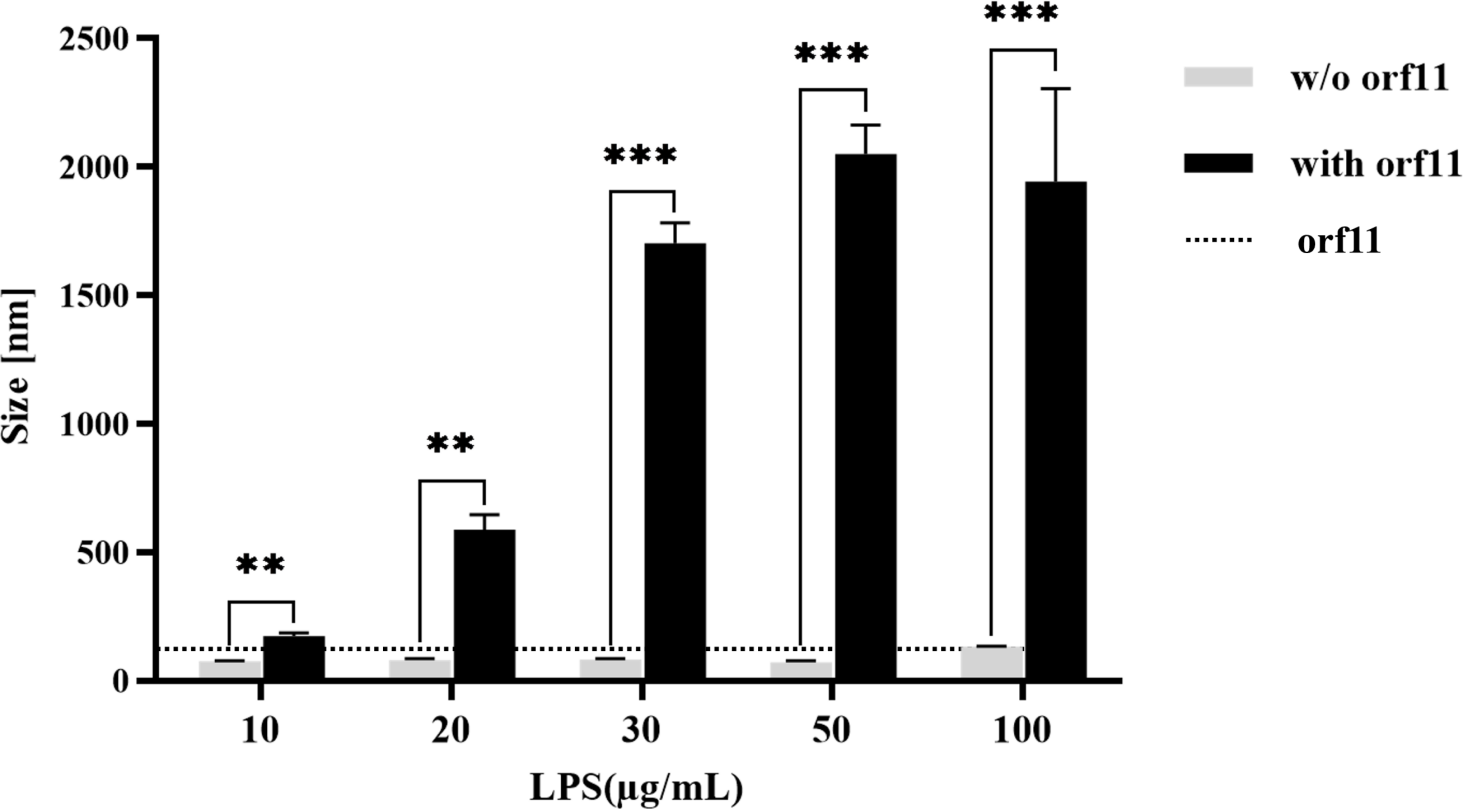
DLS analysis of orf11 protein binding to LPS at different LPS concentrations. Increasing concentrations of LPS (10–100 µg/mL) alone or with orf11 (100 µg/mL) were incubated for 30 min at room temperature and then analyzed by DLS (**P < 0.01, and ***P < 0.001, one-way analysis of variance with post hoc analyses).

Third, dynamic light scattering (DLS) was used to assess the hydrodynamic radii of particles in the presence of increasing concentrations of LPS (10–100 µg/mL), either alone or with orf11, which were incubated. LPS and orf11 particles were found to be ⁓89 and ⁓139 nm, respectively. However, after orf11 (100 µg/mL) was mixed with increasing concentrations of LPS (10–100 µg/mL), the values markedly increased (⁓174–1,943 nm, respectively; Fig. 5). These results suggest that purified orf11 protein could bind and form complexes with LPS from *M. aerodenitrificans* strain LH9 cells.

**Fig 6:**
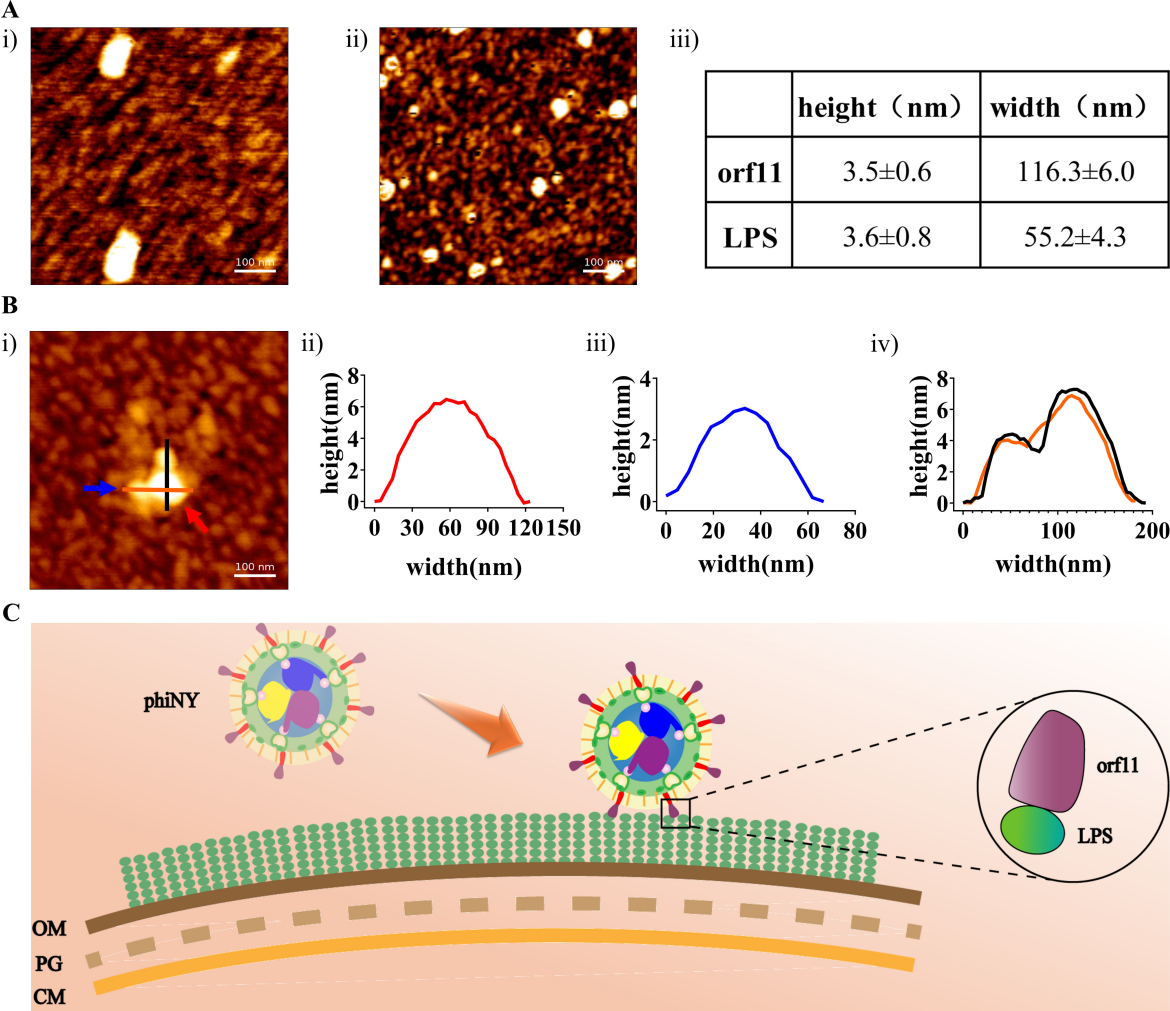
AFM imaging of the orf11, LPS, and orf11–LPS complex. (**A**) (i) image of orf11 alone, 100 µg/mL; (ii) image of LPS (20 µg/mL) alone; and (iii) mean height and width of orf11 and LPS. (**B**) (i) Image of orf11–LPS complex [5:1 (wt/wt)], with blue and red arrows indicate orf11 and LPS, respectively; (ii) height vs width of orf11; (iii) height vs width of LPS; and (iv) the black and orange straight lines indicate orf11–LPS complex. (**C**) Proposed schematic diagram of the adsorption of the host bacterium LPS by the dsRNA phage phiNY. OM, outer membrane; CM, cytoplasmic membrane; PG, peptidoglycan.

Next, atomic force microscopy (AFM) was used to visualize the complexes formed by orf11 and LPS on a mica substrate and visually observe the state of orf11 after combining with LPS (30). Figure 6Ai and ii show representative AFM images of orf11 and LPS (bright spots with irregular and oval shapes), respectively, adsorbed on mica. The mean height and width of orf11 and LPS were statistically calculated based on these bright spots, as shown in Fig. 6Aiii. Figure 6Bi shows a representative AFM image of the preincubated orf11–LPS sample adsorbed on mica. The widths of two different interacting bright spots (one at 57.5 nm and the other at 111.7 nm) are close to the widths of orf11 and LPS, respectively. Therefore, the images shown in Fig. 6Bi witness the formation of the orf11-LPS complex.

Together, our results suggest that the orf11 protein is the RBP of the dsRNA phage phiNY, and it binds specifically to LPS from *M. aerodenitrificans* strain LH9 cells as part of its adsorption strategy (Fig. 6C).

## DISCUSSION

The phage host range mainly depends on the interaction of between the RBPs and their host receptor. Studying the molecular mechanisms and evolutionary determinants of phage host range is essential for a wide variety of clinical, industrial, and biotechnological applications. Based on current knowledge, the host range of dsRNA phages is limited. Lytic dsRNA phages phi6, phi8, phi12, phi13, phi2954, phiNN, phiYY, and phiZ98 have been shown to infect the genus *Pseudomonas*, mainly including plant-pathogenic *Pseudomonas syringae* and human-pathogenic *P. aeruginosa* (15, 17–19, 21, 25, 28). Lytic dsRNA phages CAP3–CAP7 were isolated from *Acinetobacter radioresistens* (23). Interestingly, metatranscriptomic data sets found that a partial viral genome similar to the dsRNA phage genome was also detected from a pure culture of Gram-positive *Streptomyces avermitilis* (31). However, unlike other lytic dsRNA phages, dsRNA phage phiNY is the first isolated dsRNA phage from *M. aerodenitrificans* with a non-lytic life cycle (26). A key question remains: Is the adsorption strategy of dsRNA phage phiNY similar to that of other lytic dsRNA phages? Thus, in this study, we identified the RBP and host receptors of the dsRNA phage phiNY. The research results are important for understanding dsRNA phage evolution and their host range adaptation, as well as potential applications.

Our phylogenetic trees showed that orf11 of phiNY was most closely related to the P3 protein of phi2954 (Fig. 2), which uses the type IV pilus-mediated adsorption strategy (18). However, there was limited similarity (21.7%) at the amino acid sequence level. Subsequent experiments revealed that the orf11 protein binds to LPS of the host bacterium *M. aerodenitrificans* strain LH9 cells rather than the type IV pili of these cells. Thus, although orf11 of phiNY is most closely related to the P3 protein of phi2954, it uses a different adsorption strategy.

The native state of orf11 is a multimer with an approximate molecular weight of 195 kDa, according to native-PAGE and glutaraldehyde cross-linking assays (Fig. S1). In addition, the most well-described phage RBPs are homotrimers, which may reflect the essential nature of the trimeric form for binding specifically to the host receptors (2, 23, 29, 32). The correct folding of the trimeric structure of RBPs of some phages requires the assistance of phage-encoded chaperone proteins, such as *Alteromonas* Myovirus V22 and *E. coli* phage Mu’s RBPs (33, 34). The solubility of these RBPs is greatly reduced without the assistance of chaperone proteins to trimerization. Moreover, the ability of these RBPs without trimerization to recognize the host will be reduced, or they will even completely lose the ability to bind the host. Therefore, the trimeric structure of these RBPs is essential for their binding function. Currently, no reports suggest that the multimerization of RBPs of dsRNA phages requires the assistance of chaperone proteins. However, we found that the native state of orf11 is a multimer and soluble (Fig. S1 and S4). We speculate that the native state of the orf11 protein is a multimer, and this native state is important for its binding function. In addition, this requires validation using methods such as X-ray crystallography, multi-angle light scattering coupled to size exclusion chromatography, or cryogenic electron microscopy structure determination.

To establish whether the orf11 protein could bind to *M. aerodenitrificans* strain LH9 cells, we performed the whole-cell ELISA and western blotting assay using *M. aerodenitrificans* strain LH9 cells. In addition, an indirect immunofluorescence assay was used to directly observe the binding of the orf11 protein to *M. aerodenitrificans* strain LH9 cells. The results confirmed that the orf11 protein of phiNY is an RBP that binds to *M. aerodenitrificans* strain LH9 cells.

Next, to determine the surface receptor of *M. aerodenitrificans* that directly interacts with the orf11 protein, we performed three different assays. The results showed that the orf11 protein binds specifically to LPS from *M. aerodenitrificans* strain LH9 cells. In conclusion, we demonstrated that the dsRNA phage phiNY, like other lytic dsRNA phages (phi12, phi13, phiYY, and phiZ98), uses an RBP to bind to a similar host receptor (i.e., LPS) in its adsorption strategy. phiNY is a good model for elucidating the mechanism underlying non-lytic life cycles of dsRNA phages. Further studies will be needed to answer critical questions: How does the non-lytic life cycle of dsRNA phage affect the viability and adaption of host bacteria? What signals trigger entering and exiting the non-lytic life cycle of dsRNA phage?

In addition, the long-term stable persistence of phage with their host bacteria has potential applications for therapy, nanocages for gene delivery, vaccine carriers, surface disinfection, and diagnostic purposes (35–37). For example, filamentous phages (M13, f1, and fd) due to their small genome size and unique chronic life cycle to develop phage display technique, which has become a powerful tool in drug discovery, synthetic biology, nanotechnology, and many others (38). Similarly, some dsRNA phages, such as phi6 and phiNY, can also establish a non-lytic life cycle, or called a carrier state infection cycle, similar to long-term persistence infection. Thus, further research and a detailed understanding of the interaction between dsRNA phages with non-lytic life cycles and their host bacteria are beneficial to the successful exploitation of dsRNA phages with non-lytic life cycles as a powerful tool in biomedical applications (in therapy and diagnostics) and others.

## MATERIALS AND METHODS

### Bioinformatics analysis

Each ORF of the phiNY M fragment was visually inspected and compared to the ORFs of existing dsRNA phage M fragments to confirm its position in the M fragment (genes in Fig. 1 are drawn to scale and correspond among the different dsRNA phages). Amino acid sequence similarities between orf11 protein and other dsRNA phage P3 proteins were calculated using EMBOSS Needle. Nucleotide sequence similarities between orf11 and other dsRNA phage P3 genes were calculated using Clustal Omega. A phylogenetic tree was constructed using the neighborhood joining method in MEGA 11 (39) (bootstrap values are indicated at the branch points).

### Cloning, protein expression, and purification

The orf11 gene was cloned into the expression plasmid pET28a. The orf11 gene and segment thereof were PCR amplified from the cDNA fragment of phiNY. The forward and reverse primers for orf11 were 5′-CGGAATTCATGTCCAACGCTTACATC-3′ and 5′-CGAAGCTTTTACCGGGGCAC-3′. The PCR products were cloned into the multiple cloning sites of pET28a using *Eco*RI and *Hind*III restriction enzymes (the underlined primer sequences represent the restriction sites), yielding pET28a-orf11. This plasmid was transformed into *E. coli* BL21(DE3) cells, purified, and Sanger sequenced to confirm that there were no errors in the cloned DNA.

Bacteria containing the expression plasmid were cultured in 300 mL Luria–Bertani medium at 37°C until the optical density at 600 nm reached 0.6 (indicating the logarithmic growth phase). The bacteria were then cooled to 22°C, induced using 0.5 mM isopropyl-β-D-thiogalactopyranoside, and incubated overnight at 22°C to allow further protein expression. To obtain the cell lysate, centrifuged the cells (8,000 rpm, 10 min, 4°C), resuspended in PBS, disrupted by ultrasonication, and centrifuged again (12,000 rpm, 10 min, 4°C). The lysate was then mixed with Ni-NTA agarose (Qiagen), which binds to His tags, at 4°C for 1.5 h and then sequentially washed with elution buffer containing 300 mM NaCl and 50 mM NaH_2_PO_4_ plus different concentrations of imidazole (5, 10, 20, 30, 50, 60, 100, and 250 mM, pH 7.4). The purified protein was dialyzed using dialysate (20 mM Tris, 100 mM NaCl, 10% glycerol, and pH 7.4). SDS-PAGE was used to analyze whether the orf11 protein was successfully purified. The protein concentration was assessed using a Bicinchoninic Acid Protein Assay Kit (Solarbio).

### Gel electrophoresis

The purity and molecular weight of the purified orf11 protein were assessed using 12% (wt/vol) SDS-PAGE. Next, native-PAGE was conducted, involving 8% (wt/vol) separating gel and 5% (wt/vol) stacking gel (all gels and buffers were free of denaturants, and the samples were not heat treated). Gels were visualized after Coomassie Blue G-250 solution protein staining.

### Glutaraldehyde cross-linking assays

The method was carried out as previously described (34). Briefly, at room temperature, 40 µg orf11 protein mixed with 1 mM glutaraldehyde (LEAGENE) in a 120 µL reaction system was incubated for 5, 10, 20, and 30 min. Thereafter, 30 µL of the sample was added to a new tube containing 10 µL 1 M Tris-HCl (pH 8.0) for cross-linking quenching. Cross-linked orf11 was analyzed using 8% (wt/vol) SDS-PAGE.

### Enzyme-linked immunosorbent assay

In a whole-cell ELISA, LH9 strain cells cultured to the logarithmic growth phase were added to ELISA plates and allowed to evaporate overnight at 37°C. Next, 5% bovine serum albumin was used to block non-specific binding. Subsequently, 4 µg/mL purified His-tagged orf11 protein was added for 2 h. Anti-His-tag mouse antibody (ZOMANBIO) was used as the primary antibody, and horseradish peroxidase (HRP)-conjugated anti-mouse IgG (ZOMANBIO) was used as the secondary antibody. 3,3′,5,5"-Tetramethylbenzidine substrate (Solarbio) was added for color development for 30 min. The absorbance at 650 nm was determined using Cytation 5 (BioTek).

In LPS ELISA, LPS from LH9 strain or *K. pneumoniae* strain 11272018 (negative control) was added to ELISA plates and incubated overnight at 4°C. The LPS extraction steps are as follows: the same wet weight of the strain LH9 or *K. pneumoniae* strain 11272018 was collected. Then, their LPS was extracted using an LPS extraction kit (Baiaolaibo). Finally, the extracted LPS was dialyzed in the water. The standard curve of sugar concentration was plotted by the phenol-sulfuric acid method. The working concentration of LPS was 50 µg/mL.

### Western blotting assays

To verify the binding of the orf11 protein to strain LH9, western blotting analysis was performed as described previously (40). Briefly, approximately 10^8^ LH9 cells were centrifuged (2,300 g, 3 min, 4°C) in a low-bind Eppendorf tube, resuspended with 200 µL orf11 protein (100 µg/mL), and incubated at 28°C for 2 h to allow the protein to bind to strain LH9 cells. To remove unbound proteins, the strain LH9 cells were centrifuged (2,300 g, 3 min, 4°C), followed by washing twice with 1 mL PBS. Next, 35 µL 1 × SDS sample buffer was added, and the mixture was transferred to a new tube. Samples were separated by electrophoresis and then transferred to a polyvinylidene fluoride membrane. Anti-His-tag mouse antibody (ZOMANBIO) was used as the primary antibody, and HRP-conjugated anti-mouse IgG (ZOMANBIO) was used as the secondary antibody. Finally, the bands were visualized by exposing to an X-ray film after adding the enhanced chemiluminescence reagent (Meilunbio), and imaging was performed using the imaging software (Syngene). The controls were as follows: (i) strain LH9 cells alone (without orf11 protein), which led to no bands, (ii) orf11 and mCherry protein alone (without strain LH9 cells), which led to no bands, and (iii) purified orf11 and mCherry protein were used as positive controls.

### Indirect immunofluorescence assays

Strain LH9 in the logarithmic growth phase was incubated on a coverslip treated with polylysine for 30 min, blotted dry, fixed with methanol, blocked for 2 h using 5% bovine serum albumin, incubated with 20 µg/mL His-tagged orf11 protein for 2 h, incubated with monoclonal anti-His-tag mouse primary antibody (ZOMANBIO) to detect orf11, and then incubated with FITC-conjugated goat anti-mouse IgG secondary antibody (Proteintech) for 1 h in the dark. 4,6-Diamidino-2-phenylindole dihydrochloride staining solution was added to each well to stain the nuclei for 10 min in the dark at room temperature, followed by mounting with an antifade medium, drying, and examination using a confocal inverted microscope (Nikon ECLIPSE Ti).

### Affinity chromatography assays

To identify whether phiNY recognizes protein receptors on the surface of strain LH9, affinity chromatography assays were performed based on a previously described method (41). His-tagged orf11 protein was incubated with Ni-NTA agarose (Qiagen), which binds to His tags, for 2 h and then incubated with 10 mM ethanolamine for 30 min at 4°C to block sites in the agarose that did not bind to the His-tagged orf11 protein. Next, 1 mL lysate of LH9 cells in the logarithmic growth phase was mixed with the agarose and incubated overnight at 4°C to pull down host receptor proteins that may be bound by the His-tagged orf11 protein. Following washing three times with 20 mM imidazole buffer to remove proteins that bound non-specifically to the Ni-NTA agarose, the orf11 protein and the proteins that may bind to orf11 were eluted with 500 mM imidazole buffer and then analyzed by SDS-PAGE.

### DLS analysis

The particle size distribution of strain LH9 LPS at increasing doses (10–100 µg/mL) alone or with orf11 (100 µg/mL) was assessed using a NanoBrook Series Particle Analyzer (Brookhaven Instruments). orf11 protein alone was used as the control. Prior to analysis, all samples were incubated at room temperature for 30 min. All samples were diluted with deionized water. All experiments were conducted at 25°C. Each measurement was repeated five times.

### AFM imaging

AFM imaging of orf11, LPS, and orf11–LPS complex was performed using a JPK NanoWizard 4 XP (Bruker Corporation) in air-tapping mode, using silicon cantilevers, k_nom_, ~40 N/m (Bruker Corporation), and a resonant frequency of ~300 kHz. Prior to AFM imaging, orf11 protein (100 µg/mL) alone, LPS (20 µg/mL) alone, or orf11 + LPS [5:1 (wt/wt)] were incubated for 30 min, adsorbed onto mica for 10 min, washed with Milli-Q water, and dried with a stream of nitrogen.

## Data Availability

The genome sequence of orf11 is available in GenBank under accession number MW471134.1.

## SUPPLEMENTAL MATERIAL

Supplemental figures spectrum.01467-24-s0001.docx Figures S1 to S4.

ASM does not own the copyrights to Supplemental Material that may be linked to, or accessed through, an article. The authors have granted ASM a non-exclusive, world-wide license to publish the Supplemental Material files. Please contact the corresponding author directly for reuse.
