# Supplementary material for: Identification of receptor-binding protein and host receptor of non-lytic dsRNA phage phiNY
Source: Microbiol Spectr. 2024 Oct 22;12(12):e01467-24. doi: 10.1128/spectrum.01467-24 (PMC11619300; doi:10.1128/spectrum.01467-24)
Supplement: Supplemental figures — Figures S1 to S4. [file spectrum.01467-24-s0001.docx]

***Supplementary Material***

**Identification of receptor-binding protein and host receptor of non-lytic dsRNA phage phiNY**

Guoqing Ding^a,b^, Hongmei Liu^a,b^, Jing Lan^a^, Tianbao Qian^b^, Yan Zhou^a^, Tongyu Zhu^c^*, Tingting Zhang^a,b,d^*

^a^School of Public Health, the key Laboratory of Environmental Pollution Monitoring and Disease Control, Ministry of Education, Guizhou Medical University, Guiyang 561113, China

^b^Engineering Research Center of Health Medicine biotechnology of Institution of higher education of Guizhou Province，School of Biology and Engineering (School of Modern Industry for Health and Medicine), Guizhou Medical University, Guiyang, 561113, China

^c^Shanghai Medical College, Fudan University, Shanghai, 200000, China

^d^Shanghai Institute of Phage, Shanghai Public Health Clinical Center, Fudan University, Shanghai, 200000, China.

*: Address correspondence to Tingting Zhang, [ztt-gd@163.com, or](mailto:ztt-gd@163.com,%20or) Tongyu Zhu, tyzhu@fudan.edu.cn.


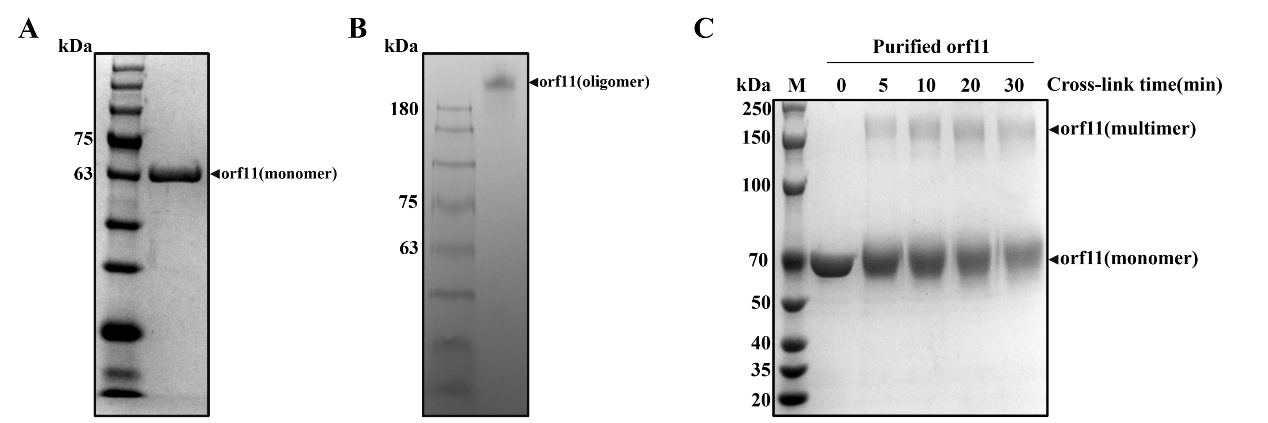


**Supplementary Figure 1**: Purified orf11 protein analyzed by SDS-PAGE, native-PAGE and glutaraldehyde cross-linking assays. (A) SDS-PAGE of purified orf11protein. The expected band of the orf11 protein monomer is indicated by a black arrow. (B) Native-PAGE of purified orf11 protein (non-denatured) revealing the naturally occurring oligomeric form of orf11.The expected band of the orf11 protein oligomer is indicated by a black arrows. (C) Glutaraldehyde cross-linking assays (incubated for 5, 10, 20, and 30 min) of purified orf11 protein revealing the oligomeric form of orf11. The expected band of the orf11 protein oligomer is indicated by a black arrow.


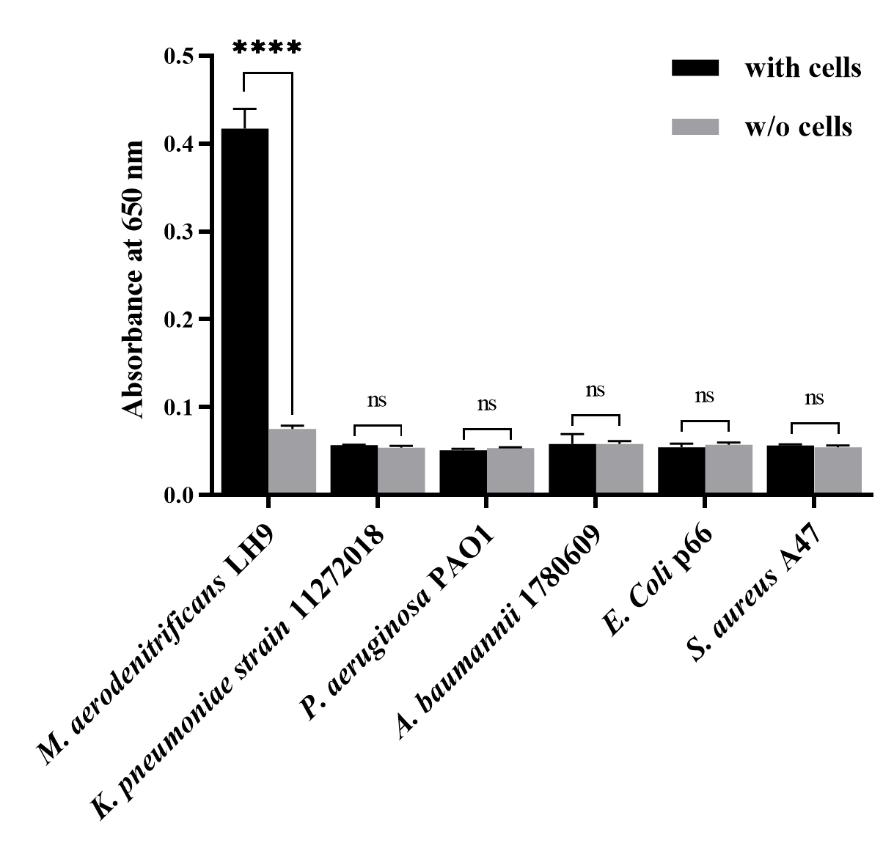


**Supplementary Figure 2**: The binding specificity of orf11 is measured by ELISA. The signal of ELISA between purified His-tagged orf11 and different strains of non-host bacteria. (****P < 0.0001, and ns, not signiﬁcant, Student’s *t* test).


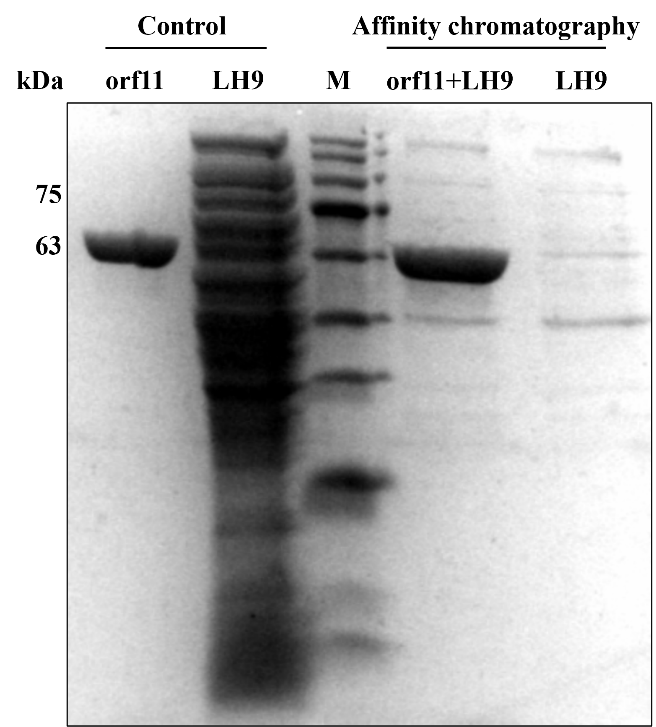


**Supplementary Figure 3**: Affinity chromatography assays assessing whether dsRNA phage phiNY targets proteins on the *M. aerodenitrificans* strain LH9 surface. Left: purified orf11 protein and whole proteins from strain LH9 cells were used as controls. Middle: molecular weight markers in lane M. Right: affinity chromatography assays; the common bands in lanes orf11+LH9 and LH9 only show faint non-specific binding to the nickel column.


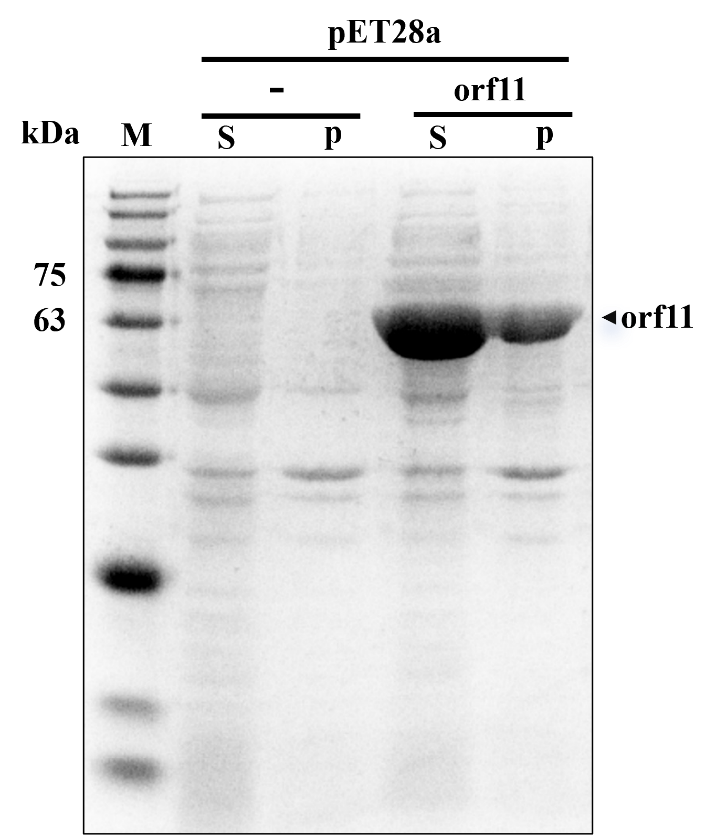


**Supplementary Figure 4:** SDS-PAGE analysis of purified orf11 protein. The empty pET28a vector as a negative control. The bands of orf11 protein are indicated by black arrowheads. S, supernatant; P, pellet.
